# Prevalence of teenage pregnancy and its associated factors in high fertility sub-Saharan Africa countries: a multilevel analysis

**DOI:** 10.1186/s12905-023-02169-7

**Published:** 2023-01-17

**Authors:** Desale Bihonegn Asmamaw, Tesfahun Zemene Tafere, Wubshet Debebe Negash

**Affiliations:** 1 Department of Reproductive Health, College of Medicine and Health Sciences, Institute of Public Health, University of Gondar, Gondar, Ethiopia; 2 Department of Health Systems and Policy, College of Medicine and Health Sciences, Institute of Public Health, University of Gondar, University of Gondar, P.O.Box 196, Gondar, Ethiopia

**Keywords:** Teenage pregnancy, Factors, Multilevel analysis, High fertility countries

## Abstract

**Background:**

Teenage pregnancies are persistently high among adolescent women in high fertility countries in sub-Saharan Africa. It has been attributed to the high unmet need for family planning in this population. The aim of this study was to determine the prevalence and factors associated with teenage pregnancy in high fertility countries in sub-Saharan Africa.

**Methods:**

Data for this study was obtained from the most recent Demographic and Health Surveys. A total weighted sample of 33,391 adolescent girls who had ever had sexual contact were included. A multilevel mixed-effect binary logistic regression model was fitted to identify the significant associated factors for teenage pregnancy. Finally, the Adjusted Odds Ratio (AOR) with a 95% confidence interval was used to declare as statistically significant.

**Results:**

The overall teenage pregnancy in sub-Saharan Africa high frtility countries was 24.88% (95% CI, 24.42, 25.35). Educational status; no formal education (AOR = 1.39, 95% CI, 1.23, 1.56) and primary education (AOR = 1.45, 95% CI, 1.30, 1.62), not working (AOR = 1.32, 95% CI, 1.21, 1.45), being married (AOR = 67.88, 95% CI, 61.33, 75.12), poor (AOR = 1.47, 95% CI, 1.32, 1.65) and middle wealth quantile (AOR = 1.21, 95% CI, 1.07, 1.35), knowledge about contracptives (AOR = 2.45, 95% CI, 2.19, 2.74), unmet need for family planning (AOR = 2.42, 95% CI, 2.14, 2.74), Angola (AOR = 9.59, 95% CI, 7.82, 11.77), Chad (AOR = 3.05, 95% CI, 2.49, 3.74), DR.Congo (AOR = 3.77, 95% CI, 3.06, 4.65), and Mali (AOR = 1.84, 95% CI, 1.47, 2.28) were factors significantly associated with teenage pregnancy.

**Conclusions:**

This study found that teenage pregnancy remains a common public health problem in the study areas. Level of education, marital status, occupation, wealth index, unmet need for family planning, knowledge about contraceptives, and country were significantly associated with teenage pregnancy. Hence, for sustainable development goal 3 to be realized by 2030, there must be investment in policy implementation and evaluation, as well as engagement with stakeholders in adolescents’ sexual and reproductive health.

## Background

Pregnancy in girls aged 10–19 years is referred to as a teen pregnancy [1]. It is estimated that 16 million girls aged 10–19 years give birth every year, accounting for almost 11% of all births worldwide [1]. Even if there is global improvement in maternal health [2], teenage pregnancy is still a common public health problem in developing countries [3] and can lead to intergenerational cycles of poverty, poor education, and unemployment [4]. Over one million babies born to teenage girls die before their first birth day, and each year 70,000 teenage girls die during pregnancy and child birth [5]. Teenage pregnancy is considered to be the leading cause of newborn and maternal mortality in developing countries [5].

Sub-Saharan Africa recorded the highest prevalence of teenage pregnancy in the world in 2013, which accounted for more than half of all births in the region, though disparities in teenage pregnancy based on location exist in sub-Saharan Africa [6]. The majority of young women 20–24 years old who gave birth before the age of 18 lived in sub-Saharan Africa, 14 out of 15 countries that had high rates of pregnancies before the age of 18 [7]. Teenage pregnancy should be considered one of the major health concerns in every health-care system because it can lead to long-term complications of physical and psychological health, deteriorate economic and social status, and be a concern from both a human rights and a public health standpoint [8, 9]. Pregnancy at an early age impacts future earning potential and leads to lifelong poverty [10]. These adverse effects persist throughout a teenager’s entire life and transfer to the next generation [11].

The World Health Organization (WHO) recognizes that investing in adolescent girls offers triple dividends through the immediate outcome during the teenage period, in their adult life, and well-being of their future children [12]. As one of the sustainable development goals (SDGs 3.1), ending preventable maternal deaths is one of the targets. The target is to reduce maternal death rates to less than 70 deaths for every 100,000 live births. Globally, by the year 2030 and preventing teenage pregnancy can help to achieve this goal since it is associated with poor maternal and child health outcomes and increased risks of dying during pregnancy and child birth [13].

However, in SSA, studies on the prevalence of teenage pregnancy and its associated factors have been extensively investigated in specific countries such as Ghana [14], Nepal [15], South Africa [16] and Ethiopia [17], studies that combine in the context of specific high fertility countries in SSA have not been carried out to understand why teenage pregnancy in those high fertility countries had a high prevalence. Additionally, there is also a multi-country study in sub-Saharan African countries [2, 18], but these previous studies failed to take into account the household/community-level factors such as community-level education, community-level media exposure, and community-level poverty and how they interact with individual-level factors to affect teenage pregnancy, which are incorporated in the current study. Therefore, this study seeks to fill this gap by assessing the prevalence and associated factors of teenage pregnancy in the high fertility sub-Saharan Africa countries using a mixed model approach. A mixed model approach will contribute to an understanding of both the individual and household/community level factors that affect teenage pregnancy and generate powerful information that triggers policy makers. Thus, the findings of this study could help policymakers, and governmental and non-governmental organizations to design programs and interventions to reduce teenage pregnancy and its complications.

## Methods

### Study settings and data source

**Fig. 1:**
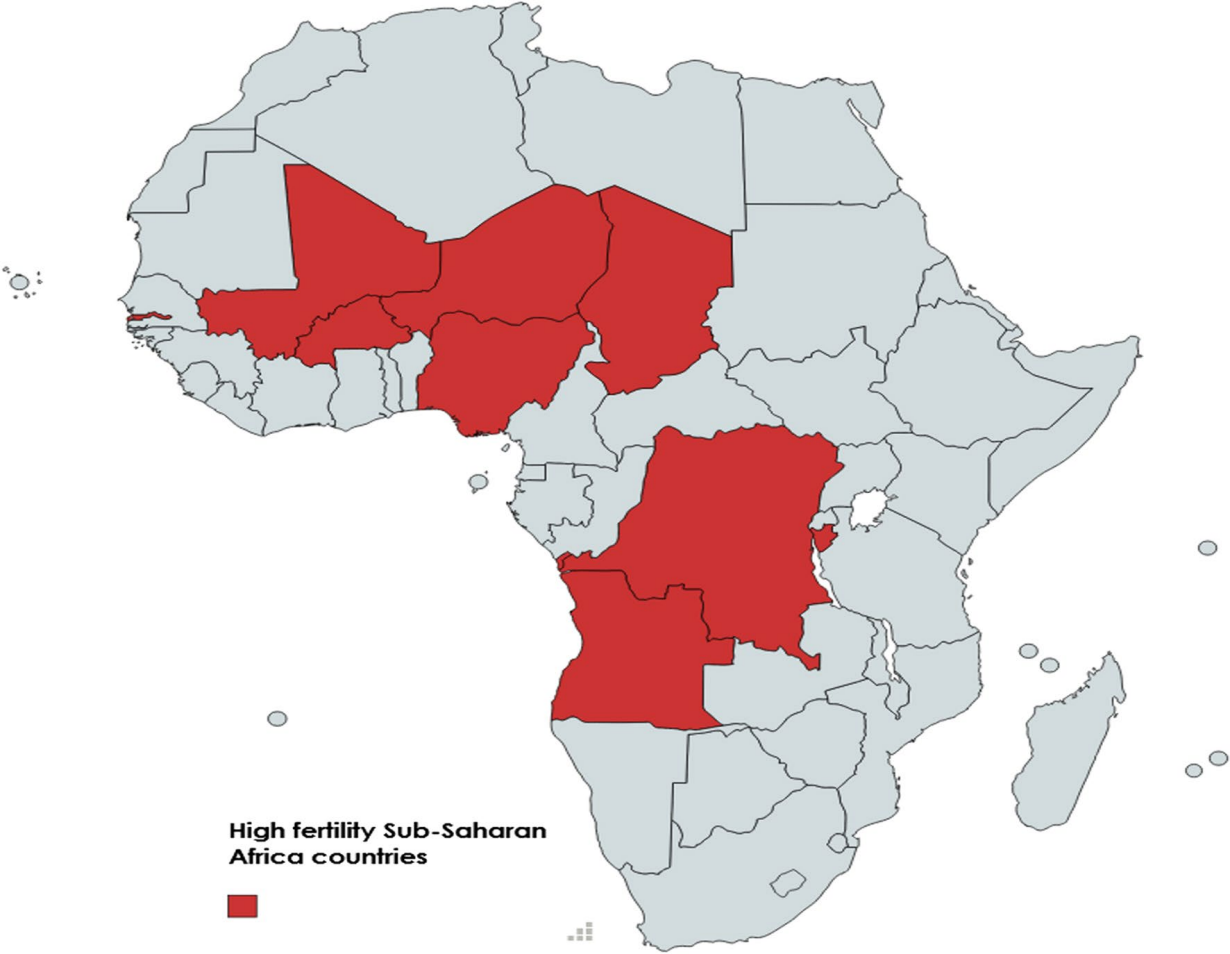
The map of the study area (high fertility Sub-Saharan Africa countries)

This study was a secondary data analysis based on the data sets from the most recent Demographic Health Surveys (DHS) conducted between January 2010 and December 2019 in high fertility countries in SSA. In this study, Niger, Democratic Republic of Congo, Mali, Chad, Angola, Burundi, Nigeria, Gambia, and Burkina Faso were included. These countries were selected because they are the top ten countries with high fertility rates in SSA, with fertility rates above 5.0, a value that is higher than the rate of 4.44 in Africa and 2.47 worldwide [19]. One country (Somalia) with no DHS data was excluded from the analysis (Fig. 1).

DHS is a nationally representative household survey that has been conducted across low- and middle-income countries every five years [20]. It has been an essential data source on issues of reproductive health in low and middle-income countries as it gathers data on a number of reproductive health issues [20].

In the current study, we used the woman’s record (IR file) data set and extracted the dependent and independent variables. A two-stage stratified sampling technique was used to select the study participants. All the adolescent girls aged 15–19 years and those who had ever had sex (a total weighted sample of 33,391) were considered for the final analysis. Detailed information on the survey country and the number of adolescent girls in each selected country was provided (Table 1).

**Table 1:** Description of Surveys and sample size characteristics in high fertility countries in SSA (n = 33,391)

Countries	Survey year	Weighted sample(n)	Weighted sample (%)
Angola	2015/16	3444	10.31
Burkina Faso	2010	3312	9.92
Burundi	2016/17	3859	11.56
Chad	2014/15	3934	11.78
DR Congo	2013/14	4053	12.14
Gambia	2013	2407	7.21
Mali	2018	2104	6.30
Nigeria	2012	8448	25.30
Niger	2012	1830	5.48

### Variables of the study

#### Dependent variable

The dependent variable for this study was getting pregnant during the age of 15–19 years among adolescents who had ever had sex. A woman was considered to be experiencing a teenage pregnancy if her age was between 15 and 19 years, and if she had ever been pregnant before or during the survey. The dependent variable was derived using the variables: the number of women who have had a birth and the number of women who had not gave birth but are pregnant at the time of the interview [21].

#### Explanatory variables

The explanatory variables considered for the current study were both individual and community-level factors. The individual-level variables include the following; educational status of the women, marital status, occupation of the women, wealth index, knowledge about contraceptives, and unmet need for family planning. The community-level variables were community women’s education, community level poverty, community level media exposure, residence, and country. In DHS, except for residence and country, all the other factors were collected at the individual level. Hence, we generate three community-level factors such as community-level women’s education, community-level poverty, and community-level media exposure by aggregating the individual-level factors at the cluster level and categorizing them as high and low based on the median value (Table 2).

**Table 2:** Description and measurements of independent variables

Variables	Description
Women education level	No formal education, Primary education, and Secondary education and higher
Place of residence	Rural, Urban
Women occupation	Re-coded in two categories not working and Working
Partner education level Marital status	No formal education, primary education, and secondary education and higher Recoded in two categories unmarried and married
Wealth index Knowledge about contraceptives unmet need for family planning	It was categorized as Poor, Middle and Rich Not know any methods, Knows traditional methods, and Knows modern methods Yes, No
Media exposure	Yes, No
Covered by health insurance	Yes, No
Community level media exposure	High, Low
Community level education	High, Low
Community level poverty	High, Low

#### Data management and analysis

Stata version 14 software was used for data analysis. The data were weighted throughout the analysis to ensure the DHS sample's representativeness and to obtain reliable estimates and standard errors. Descriptive statistics were described using frequencies, percentages, median, and interquartile range, and were presented using tables, figures, and narratives. We checked the Interclass Correlation Coefficient (ICC) and Median Odds Ratio (MOR) to determine whether there was clustering or not. Four models were fitted in this study: the null model, which had no independent variables, the model I, which had individual-level factors, model II, which had community-level factors, and model III, which had both individual and community-level components. Deviance was used to assess the model fitness, and the model with the lower deviance (model III) was the best-fitted model. Variables with a p-value less than 0.2 in the bivariable analysis were used for multivariable analysis. Finally, in the multivariable analysis, adjusted odds ratios with 95% confidence intervals and a p-value of less than 0.05 were used to identify associated factors for teenage pregnancy.

## Results

### Individual level factors

A total of 33,392 weighted adolescent girls were included in this analysis. Nearly one-quarter (22%) of the women were aged 18 years. Most (79.03%) of the household heads were males. Of the study participants, majority (96.04%) did not use contraceptives (Table 3).

**Table 3:** Individual characteristics of study participants in high fertility countries in sub-Saharan Africa (n = 33,392)

Variables	Category	Frequency	Percentage
Age in years	15	7252	21.72
	16	6635	19.87
	17	6407	19.19
	18	7614	22.80
	19	5484	16.42
Educational status of participants	No formal education	9421	28.21
	Primary education	8304	24.87
	Secondary & Higher education	15,667	46.92
Marital status	Married	9047	27.09
	Not married	24,345	72.91
Occupation of participants	Not working	14,925	44.70
	Working	18,467	55.30
Wealth status	Poor	11,538	34.55
	Middle	6479	19.40
	Rich	15,375	46.04
Mass media exposure	Yes	21,577	64.62
	No	11,814	35.38
Sex of household head	Male	26,390	79.03
	Female	7002	20.97
Contraceptive use	Yes	1323	3.96
	No	32,069	96.04
Unmet need for family planning	Yes	2736	8.19
	No	30,656	91.81
Knowledge about contraceptive methods	Knows about modern methods	26,700	79.96
	Knows about traditional methods	212	0.64
	Do not know any methods	6479	19.40

### Community- level characteristics of the participants

Of the study participants, three-fifth (60.88%) were rural dwellers. Half (50.24%) of the adolescent girls were from communities with low proportion of media non-exposed. Nearly half (47.46%) of the adolescent girls were from communities with high proportion of poor people (Table 4).

**Table 4:** community level characteristics of study participants (n = 33,392)

Variables	Category	Frequency	Percentage
Place of residence	Urban	13,063	39.12
	Rural	20,329	60.88
Community education status	Low proportion of non-educated	15,680	46.96
	High proportion of non-educated	17,712	53.04
Community media exposure	Low proportion of non-exposed	16,775	50.24
	High proportion of non-exposed	16,617	49.76
Community wealth status	Low proportion of poor	17,543	52.54
	High proportion of poor	15,849	47.46

### Prevalence of teenage pregnancy

**Fig. 2:**
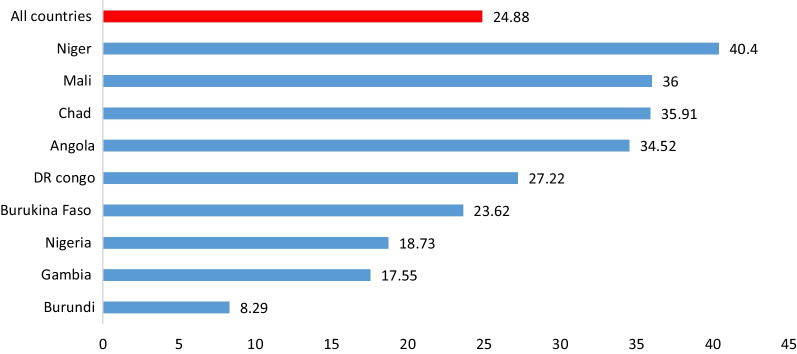
Magnitude of adolescent girls pregnancy in high fertility SSA countries

The overall prevalence of adolescent girls’ pregnancy in the high fertility countries was 24.88 (95% CI, 24.42, 25.35). The lowest teenage pregnancy was in Burundi (8.29%) whereas, Niger accounts the highest prevalence (40.4%) of teenage pregnancy (Fig. 2).

## Random effect (measures of variation)

The intra-class correlation coefficient in the null model was 0.095, which indicates that the 9.5% variation among the teenage pregnancy was due to differences between clusters. In addition to this, the median odds ratio (1.75) in the null model implies that teenagers within a higher cluster of teenage pregnancy had 1.75 times higher chance of teenage pregnancy than teenagers from lower cluster of teenage pregnancy if selected randomly from two different enumeration areas. With regard to the proportionate change in variance (PCV), 45.71% of the variability was explained by the final model. The third model was selected as the final model since it has the lowest deviance (18,954.18) (Table 5).

**Table 5:** Multivariable analysis of factors associated with teenage pregnancy in high fertility SSA countries

Variables	Null model	Model 1 AOR (95% CI)	Model 2 AOR (95%CI)	Model 3 AOR (95%CI)
Individual level Characteristics				
Educational status of participants				
No formal education		0.95 (0.86, 1.06)		1.39 (1.23, 1.56)*
Primary education		1.49 (1.34, 1.64)		1.45 (1.30, 1.62)*
Secondary and higher		1		1
Occupation				
Not working		1.05 (0.97, 1.14)		1.32 (1.21, 1.45)*
Working		1		1
Marital status				
Married		52.9 (48.35, 57.89)		67.88(61.33, 75.12)*
Unmarried		1		1
Wealth index				
Poor		1.45 (1.32, 1.59)		1.47 (1.32, 1.65)*
Middle		1.19 (1.07, 1.32)		1.21 (1.07, 1.35)*
Rich		1		1
Knowledge about contraceptives				
Knows modern methods		1.74 (1.57, 1.92)		2.45 (2.19, 2.74)*
Knows traditional methods		1.58 (1.02, 2.44)		1.41 (0.90, 2.19)
Not know any methods		1		1
Unmet need				
Yes		3.23 (2.85, 3.65)		2.42 (2.14, 2.74)*
No		1		1
Community level variables				
Community level poverty				
High			1.12 (1.01, 1.23)	0.91 (0.80, 1.03)
Low				1
Community media exposure				
Low			0.85 (0.77, 0.94)	0.94 (0.84, 1.07)
High			1	1
Residency				
Rural			2.33 (2.18, 2.50)	0.95 (0.85, 1.07)
Urban			1	1
Community level education				
Low			1.44 (1.31, 1.59)	1.01 (0.89, 1.14)
High			1	1
Country				
Angola			3.05 (2.66, 3.51)	9.59 (7.82, 11.77)*
Burkina Faso			1.20 (1.04, 1.39)	0.88 (0.72, 1.09)
Burundi			0.29 (0.25, 0.34)	1.22 (0.96, 1.55)
Chad			2.19 (1.91, 2.51)	3.05 (2.49, 3.74)*
DR Congo			1.68 (1.46, 1.93)	3.77 (3.06, 4.65)*
Mali			2.24 (1.93, 2.60)	1.84 (1.47, 2.28)*
Nigeria			1.04 (0.91, 1.19)	1.16 (0.95, 1.42)
Niger			2.55(2.19, 2.98)	1.10 (0.89, 1.37)
Gambia			1	1
Random effect				
Variance	0.35	0.25	0.21	0.19
ICC (%)	9.5	7.1	6.1	5.8
MOR	1.75	1.60	1.54	1.51
PCV (%)	Ref.	28.57	40	45.71
Model fitness				
Deviance	36,775.74	202,000.52	34,189.22	18,954.18

^*^Statistically significant at *p*-value < 0.05, *AOR* Adjusted odds ratio, *COR* Crude odds ratio

Null model: adjusted for individual-level characteristics, Model 2: Adjusted for community-level characteristics, Model 3: adjusted for both individual and community-level characteristics

## Factors associated with teenage pregnancy

In the final model, adolescent girls education, women occupation, marital status, wealth index, knowledge of modern contraceptives, unmet need, and country were significantly associated with teenage pregnancy.

Accordingly, the odds of teenage pregnancy among adolescent girls who had not learned formal education and completed primary education were 1.39 (AOR = 1.39, 95% CI, 1.23, 1.56), and 1.45 (AOR = 1.45, 95% CI, 1.30, 1.62) times higher than those who had completed secondary and higher education, respectively.

The odds of experiencing a teenage pregnancy among adolescent girls who had work was 1.32 (AOR = 1.32, 95% CI, 1.21, 1.45) times higher than their counterparts.

Like wise, the odds of experiancing teenage pregnancy among married adolescent girls were 67.88 (AOR = 67.88, 95% CI, 61.33, 75.12) times higer than unmarried adolescent girls.

The odds of teenage pregnancy among poor, middle adolescent girls was 1.47 (AOR = 1.47, 95% CI, 1.32, 1.65) and 1.21 (AOR = 1.21, 95% CI, 1.07, 1.35) times higher than those rich adolescent girls, respectively.

Moreover, adolescent girls who had knowledge about modern contraceptive methods were 2.45 times more likely to become pregnant as compared with those who had not have knowledge about modern contraceptive methods (AOR = 2.45, 95% CI, 2.19, 2.74).

Those adolescent girls who had unmet need were 2.42 (AOR = 2.42, 95% CI, 2.14, 2.74) times more likely to experience pregnancy than those adolescent girls who had met need.

The odds of teenage prgnancy among countries of Angola, Chad, DR. Congo, Mali was 9.59, 3.05, 3.77, 1.84 (AOR = 9.59, 95% CI, 7.82, 11.77), (AOR = 3.05, 95% CI, 2.49, 3.74), (AOR = 3.77, 95% CI, 3.06, 4.65), (AOR = 1.84, 95% CI, 1.47, 2.28) times higher than Gambia, respectively (Table 5).

## Discussion

This study aimed to assess the prevalence and associated factors of teenage pregnancy in high-fertility countries in sub-Saharan Africa. Accordingly, one in four adolescent girls experiences pregnancy. Factors such as women’s education, occupation, marital status, wealth quantile, knowledge about modern contraceptive methods, unmet needs for family planning, and country were independently associated with teenage pregnancy.

In this study, the overall prevalence of teenage pregnancy was 24.88%, which is higher than the studies conducted in Ethiopia 23.59% [3], Cameroon 8.7% [22], Kenya 23.3% [23], Nigeria 22.9% [24] and South Africa 19.2% [25]. This might be because of the large sample size and the fact that we included participants from different countries with a wide variety of socioeconomic status and cultural norms. Besides, the possible justification might be that in high fertility countries, there is a high proportion of early sexual initiations and early marriages [26, 27]. These factors may contribute to the high prevalence of teenage pregnancy in the present study as compared to the previous one.

However, the current finding is lower than studies conducted in East Africa 54.6% [28]. The possible justification for this difference might be due to differences in socio-demographic characteristics among participants. For instance, the proportion of adolescent girls who had no formal education in this study was 28.21%. Whereas in the East Africa study, the figure was 8.28%. Moreover, the proportion of adolescent girls who were married in the current study was 27%, while in the previous study it was 46.46%. In this regard, previous research has documented that educational status has a negative association with teenage pregnancy, whereas marital status has a positive association with teenage pregnancy [6, 18, 29, 30]. Hence, preventing early marriage and having a large proportion of adolescents with secondary education or higher may reduce the odds of teenage pregnancy.

This study showed an association between education and teenage pregnancy. The odds of teenage pregnancy was 1.39 and 1.45 times higher among adolescents who had no formal education and primary education compared to secondary/ higher education levels, respectively. This is in line with studies conducted in East Africa [31] and sub-Saharan Africa [6]. The possible reason could be that education enhances women’s autonomy and decision-making power to negotiate with their partners about sexual and reproductive rights. It also enhances economic independence, leading to the postponement of early marriage and a reduction of fertility [31]. This indicates that educational status has a great impact on preventing teenage pregnancy.

In the current study, adolescent girls who have no work had higher odds of teenage pregnancy compared with those who had work. This finding is in line with a study conducted in Nepal [32]. This might be explained by the fact that adolescents with no work might have no opportunity to access contraceptives and might be engaged in early sexual intercourse for the purpose of their economic benefit. Furthermore, adolescent girls who had work can financially support a poor family and stay in the family for a longer period without marrying. However, this finding is inconsistent with study done in sub-Saharan Africa [6].

Married adolescent girls had higher odds of being pregnant compared to their counterparts, which is supported by studies done in East Africa [31] and sub-Saharan Africa [6]. This might be as adolescent girls married, being more likely to be exposed to frequent and unprotected sexual activity, often leading to an early and risky first birth. In developing countries, fertility was proved within a year of marriage [33]. Furthermore, their husband's influence on them not to use contraception may be a factor [34]. The finding of this study suggest that investment in ending early marriage is crucial to reducing teenage pregnancy and its complications.

This multilevel analysis found that household economic status significantly influenced the odds of teenage pregnancy in which the odds of teen age pregnancy was significantly higher among adolescents from poor and middle class households compared to the rich household class, which is in line with a study done in East Africa [31]. This could be because adolescent girls from higher poverty may be exposed to early marriage and sexual initiation and can not afford the cost of sexual and reproductive health services, including contraceptives [35]. Moreover, adolescent girls from poor households may be involved in transactional sex as an economic survival strategy, and this leads to pregnancy at a younger age [6].

Adolescent girls who had higher knowledge of contraceptive methods were more likely to have teenage pregnancies compared to their counterparts. Despite being counter-intuitive. This might be due to adolescent girls’ being aware of modern contraceptive methods after pregnancy occurs. Another possible reason could be that pregnancy might have occurred despite knowledge of family planning because of social pressure or desire to become pregnant and was not mitigated by outside incentives to delay pregnancy [36]. Studies from the SSA have found that higher knowledge of modern contraceptive methods, particularly among adolescents, does not always lead to higher contraceptive use [37, 38]. Adolescent girls from Angola, Chad, the Demographic Republic of Congo, and Mali had a higher chance of being pregnant early.

Furthermore, the unmet need for family planning was significantly associated with teenage pregnancy. Women with unmet needs for family planning had higher odds of experiencing teenage pregnancy than those who met their family planning needs. The possible justification might be that the unmet need for family planning exposes adolescents to the risk of unintended pregnancy [39, 40]. Hence, these findings indicate that addressing the unmet need for family planning among adolescents is a good opportunity to reduce teenage pregnancy in the study areas so far.

### Strength and limitation of the study

The study's main strength was that it used a large sample size of nationally representative survey data from high fertility countries in SSA. We employed multilevel analysis (an advanced model) to accommodate the hierarchical nature of the data and to get a reliable estimate and standard error. However, the data used in this study were cross-sectional, and we were unable to reveal the temporal relationship between teenage pregnancy and explanatory variables. Moreover, DHS surveys are based on self-reported information, and they might have the possibility of recall bias.

## Conclusion

This study found that teenage pregnancy remains a common public health problem in the study area. Level of education, marital status, occupation, wealth index, unmet need for family planning, knowledge of contraceptives, and country were associated with teenage pregnancy in high fertility countries in sub-Saharan Africa. Hence, for sustainable development goal 3 to be realized by 2030, there must be an investment in policy implementation and evaluation, as well as engagement with stakeholders in adolescents’ sexual and reproductive health. Furthermore, designing interventions targeting higher risk groups such as those from the poor household class through promoting maternal education and empowerment is crucial to reducing adolescent pregnancy.

## Data Availability

Data for this study were sourced from Demographic and Health surveys (DHS), which is freely available online at https://dhsprogram.com.

## Abbreviations

AOR: Adjusted odds ratio
CI: Confidence interval
DHS: Demographic and health survey
ICC: Intra-class correlation coefficient
MOR: Median odds ratio
PCV: Proportional change in variance
SSA: Sub-Saharan Africa
WHO: World Health Organization
